# Hypothermia and heart rate variability in a healthy newborn piglet model

**DOI:** 10.1038/s41598-022-22426-3

**Published:** 2022-10-31

**Authors:** Mette Vestergård Pedersen, Ted Carl Kejlberg Andelius, Hannah Brogård Andersen, Kasper Jacobsen Kyng, Tine Brink Henriksen

**Affiliations:** 1 Department of Pediatrics, Aarhus University Hospital, Palle Juul-Jensens Blvd. 99, Aarhus N, Denmark; 2 Department of Clinical Medicine, Aarhus University, Palle Juul-Jensens Blvd. 99, Aarhus N, Denmark

**Keywords:** Paediatric research, Biomarkers

## Abstract

Decreased heart rate variability (HRV) may be a biomarker of brain injury severity in neonatal hypoxic-ischemic encephalopathy for which therapeutic hypothermia is standard treatment. While therapeutic hypothermia may influence the degree of brain injury; hypothermia may also affect HRV per se and obscure a potential association between HRV and hypoxic-ischemic encephalopathy. Previous results are conflicting. This study aimed to investigate the effect of hypothermia on HRV in healthy, anaesthetised, newborn piglets. Six healthy newborn piglets were anaesthetised. Three piglets were first kept normothermic (38.5–39.0 °C) for 3 h, then exposed to hypothermia (33.5–34.5 °C) for 3 h. Three piglets were first exposed to hypothermia for 3 h, then rewarmed to normothermia for 3 h. Temperature and ECG were recorded continuously. HRV was calculated from the ECG in 5 min epochs and included time domain and frequency domain variables. The HRV variables were compared between hypothermia and normothermia. All assessed HRV variables were higher during hypothermia compared to normothermia. Heart rate was lower during hypothermia compared to normothermia and all HRV variables correlated with heart rate. Hypothermia was associated with an increase in HRV; this could be mediated by bradycardia during hypothermia.

## Introduction

Neonatal encephalopathy, which may be caused by hypoxia and ischemia, is the most common cause of long-term disability in term infants^1^. A perinatal hypoxic-ischemic event may affect brain function and if specific clinical criteria are present the condition is called hypoxic-ischemic encephalopathy^1^. Early biomarkers for diagnosis, prognosis, and effect of treatment on brain injury in hypoxic-ischemic encephalopathy are warranted^2^.

Heart rate variability (HRV) is a measure of variation in time interval between heart beats. It is considered a reflection of output from the autonomous nervous system^3^. Thus, HRV has been suggested as a novel early biomarker of severity of brain injury in newborns with hypoxic-ischemic encephalopathy^4–6^. Preclinical and clinical studies have found that low HRV was associated with death or more severe brain injury measured by electroencephalogram, magnetic resonance imaging, and worse neurodevelopmental outcome on Griffiths development scales^7–11^.

Neonates with moderate to severe hypoxic-ischemic encephalopathy are treated with hypothermia to a target body-temperature of 33.5 to 34.0 °C for 72 h. While this treatment ameliorates the degree of brain injury and improves neurodevelopmental outcome, hypothermia also affects the autonomous nervous system^12^. This can be seen as bradycardia, prolonged QT-interval with subsequent cardiac arrythmias, and haemodynamic changes including increased peripheral vasoconstriction during hypothermia^12–14^. Thus, hypothermia may affect HRV per se and consequently obscure the association between hypoxic-ischemic encephalopathy and HRV.

The effect of hypothermia on HRV has previously been investigated in clinical studies of neonates with hypoxic-ischemic encephalopathy but results are conflicting^15–17^. These studies examined HRV in neonates with hypoxic-ischemic encephalopathy. Thus, the influence of temperature on HRV may reflect the degree of brain injury, or result from confounding by other factors related to HRV, such as gestational age at birth, birthweight, presence of infections, seizures, or sedation which was not controlled for in all studies^4,6^.

For ethical reasons it is impossible to study the effect of temperature on HRV in healthy newborn babies. This can instead be done in an animal model where it is also possible to control for some of the previous mentioned factors related to HRV. Newborn piglets have previously been used to investigate various neonatal diseases, treatments, and physiological changes after birth^18–20^. Thus, the aim of this study was to investigate the effect of hypothermia on HRV in a controlled standardized setting in healthy, anesthetized, newborn piglets.

## Results

Six animals, four females and two males, completed the experimental procedures. There were no unexpected or adverse events. Blood gas variables (pH, pCO_2_, haemoglobin, blood-glucose, lactate, standard base-excess, and mean arterial blood pressure (MABP) were comparable between animals during normothermia and hypothermia (Table 1). PO_2_ was higher during normothermia compared to hypothermia (Table 1). In Animal 1 electrocardiogram (ECG) data was only available for HRV analysis for 1 h during normothermia and 1 h during hypothermia. In Animal 2 ECG data was only available for HRV analysis for 1 h during hypothermia and 3 h during normothermia. The number of ECG epochs analysed from each animal was comparable during normothermia and hypothermia (Table 1).

**Table 1:** Number of analyzed epochs from each animal, blood-gas measures, mean arterial blood pressure, and changes in temperature in six healthy, anaesthetised, newborn piglets exposed to 3 h of normothermia (38.5–39.5 °C) and 3 h of hypothermia (33.5–34.0 °C).

	Normothermia	Hypothermia
Epochs	35 (29–36)	31 (6–37)
pH	7.51 (0.02)	7.52 (0.05)
pCO_2_	5.2 (0.3)	4.9 (0.7)
pO_2_	15.6 (1.2)*	14.0 (1.8)*
Hemoglobin	5.6 (0.3)	5.6 (0.3)
Glucose	6.5 (5.6–9.2)	6.3 (5.8–8.7)
Lactate	1.2 (0.2)	1.0 (0.3)
Base excess	8.3 (2.4)	7.4 (2.3)
MABP	52 (5)	49 (4)
∆T	0.2 (0.1–0.4)	0.2 (0.1–0.5)

Blood-gas measures and mean arterial blood pressure were measured every hour. Changes in temperature are during 15 min.

Data presented as mean (± SD) or median (IQR).

*MABP* mean arterial blood pressure, *∆T* 15 min change in temperature.

N = 5 for blood gas values, N = 6 for MAP and ∆T.

*p < 0.05 compared with paired t-test or Wilcoxon test.

### HRV

**Figure 1:**
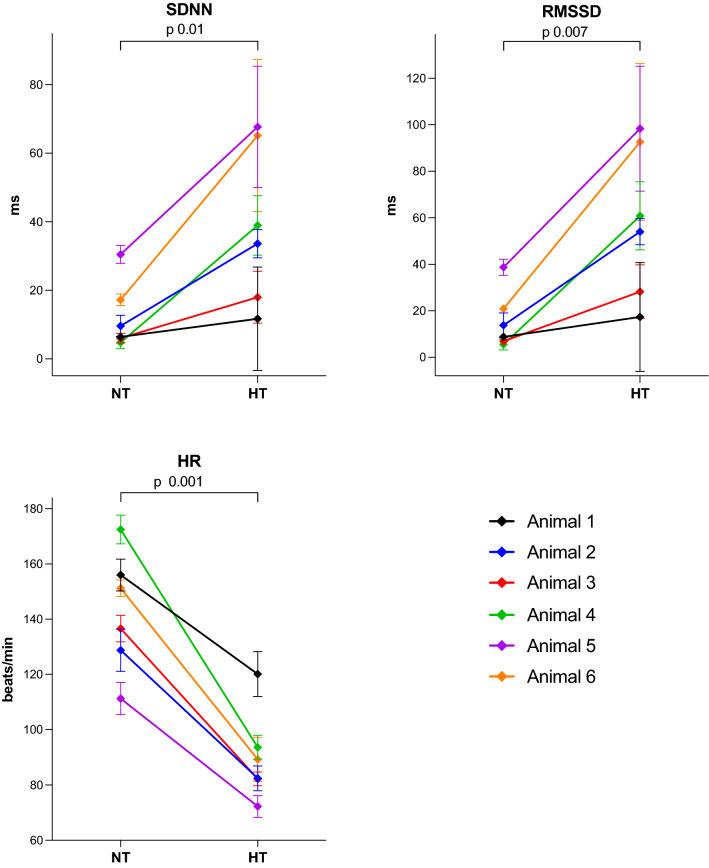
HRV time domain variables and heart rate in six healthy, anaesthetised, newborn piglets exposed to 3 h of normothermia (38.5–39.5 °C) and 3 h of hypothermia (33.5–34.0 °C). Data are presented as mean with SD of 3 h of normothermia (NT) and mean with SD of 3 h of hypothermia (HT). *P*-value comparing NT with HT with paired t-test. NT: Normothermia; HT: Hypothermia; SDNN: Standard deviation of normal-to-normal interval; RMSSD: Root mean square of successive differences; logVLF: log-transformed power in the very low frequencies; logLF: log-transformed power in the low frequencies; logHF: log-transformed power in the high frequencies; HR: heart rate.

**Figure 2:**
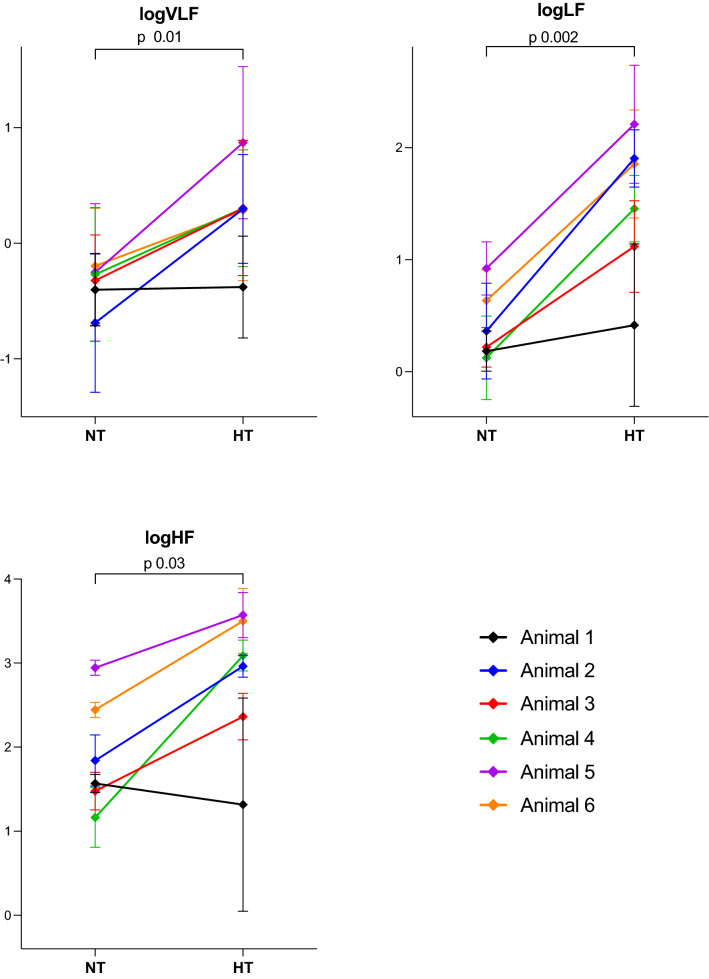
HRV frequency domain variables in six healthy, anaesthetised, newborn piglets exposed to 3 h of normothermia (38.5–39.5 °C) and 3 h of hypothermia (33.5–34.0 °C). Data are presented as mean with SD of 3 h of normothermia (NT) and mean with SD of 3 h of hypothermia (HT). *P*-value comparing NT with HT with paired t-test. NT: Normothermia; HT: Hypothermia; SDNN: Standard deviation of normal-to-normal interval; RMSSD: Root mean square of successive differences; logVLF: log-transformed power in the very low frequencies; logLF: log-transformed power in the low frequencies; logHF: log-transformed power in the high frequencies; HR: heart rate.

The time domain variables standard deviation of normal-to-normal-interval (SDNN) and root mean square of successive differences (RMSSD) were significantly higher during hypothermia compared to normothermia (Fig. 1). The frequency domain variables of absolute power in bands of very low frequencies (VLF), low frequencies (LF), and high frequencies (HF) were also significantly higher during hypothermia compared to normothermia (Fig. 2). The increase in the HRV variables from normothermia to hypothermia was not as pronounced for Animal 1 as for the other animals (Fig. 1 and Fig. 2). This animal also had lower HF during hypothermia than during normothermia.

**Figure 3:**
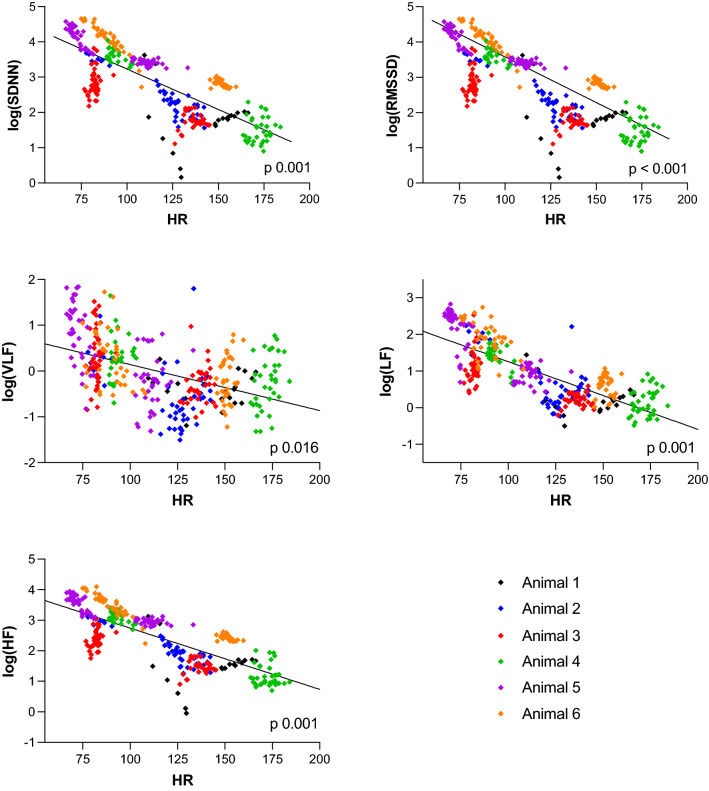
Clustered linear regression of heart rate and HRV measures in six healthy, anaesthetised, newborn piglets exposed to 3 h of normothermia (38.5–39.5 °C) and 3 h of hypothermia (33.5–34.0 °C). SDNN: Standard deviation of normal-to-normal interval; RMSSD: Root mean square of successive differences; log(VLF): log-transformed power in the very low frequencies; log(LF): log-transformed power in the low frequencies; log(HF): log-transformed power in the high frequencies; HR: heart rate.

Heart rate was significantly lower during hypothermia compared to normothermia (Fig. 1). All HRV variables decreased significantly with increasing heart rate (Fig. 3).

The effect of hypothermia on HRV and heart rate remained statistically significant when adjusting for initial temperature, I.e., whether the animal was first exposed to hypothermia and then rewarmed or vice versa in a linear multiple regression model. In this model, F-test on temperature as categorical variable (hypothermia or normothermia) showed that temperature contributed significantly to the model for all HRV variables (SDNN F=0.01, RMSSD F=0.01, VLF F=0.004 LF F=0.001 HF F=0.005).

Fluctuations in temperature were similar during hypothermia and normothermia (Table 1). We found no correlation between changes in temperature during hypothermia and any HRV variables (data not presented, p-values of linear regressions: SDNN p=0.4, RMSSD p=0.4, VLF p=0.1, LF p=0.5, HF p=0.4).

## Discussion

We found that hypothermia (core temperature of 33.5 to 34.0 °C) causes high HRV compared to normothermia (core temperature of 38.5 to 39.5 °C) in healthy anaesthetized newborn piglets.

Several mechanisms may explain the association between HRV and temperature. Hypothermia has been shown to cause bradycardia, reduce myocardial energy and metabolic demands^14,21^. HRV has been suggested to be reversely associated with heart rate^22,23^. Consequently, bradycardia during hypothermia would cause high HRV. We add to this by demonstrating an inverse relationship between all selected HRV variables and heart rate, thus suggesting the increased HRV during hypothermia to be mediated by bradycardia.

Low heart rate during hypothermia could be a direct effect of hypothermia on the sinus node and the myocardium. In a model of isolated rat hearts reduction of temperature caused high HRV (triangular interpolation of the mode, triangular index and frequency domain variables) suggesting that HRV may be mediated by cardiac factors and factors related to the central nervous system^24^. As the time domain variables depend on the number of observations the reduction in number of inter-beat time-interval-observations during bradycardia will cause high SDNN and RMSSD^3^. Therefore, considering heart rate when evaluating HRV is relevant.

Homeostasis of body temperature relies on a physiological feed-back system with vasoconstriction and vasodilatation^25^. Exogenous temperature instability would affect vascular resistance and thus the autonomic nervous system. This dynamic response from the autonomic nervous system to temperature instability may be visualized by HRV^3^. Thermoregulation is relatively slow and would thus be revealed in the frequency variables VLF and LF^21^. We found fluctuations in temperature during hypothermia and normothermia that were similar and no correlation between fluctuations in temperature during hypothermia and HRV.

In previous studies in neonates with hypoxic-ischemic encephalopathy there was significant effect of temperature on some but not all of the HRV variables investigated in the present study^15–17^. Massaro et al. found HRV to be dependent on temperature with higher LF during hypothermia in neonates with hypoxic-ischemic encephalopathy^15^. However, the brain injury in hypoxic-ischemic encephalopathy develops over time and consequently, HRV is dynamic over time; it may be increased in the early hours after a hypoxic-ischemic event and decreased later on^10,26^. Timing of HRV measurement may thus influence the results by Massaro et al. The dynamics of HRV in term neonates with hypoxic-ischemic encephalopathy remains to be fully understood. Goulding et al. found HF to be higher in neonates with moderate hypoxic-ischemic encephalopathy undergoing hypothermia treatment compared to neonates with moderate hypoxic-ischemic encephalopathy and no hypothermia treatment^17^. The degree of brain injury in neonates with moderate hypoxic-ischemic encephalopathy may differ due to the therapeutic effect of hypothermia. A milder brain injury would cause higher HRV. Thus, Vesoulis et al.^15^ suggested low HRV during normothermia compared to hypothermia in neonates to be caused by withdrawal of the therapeutic effect of hypothermia on brain injury and not by temperature per se. We demonstrate in this study that the neurophysiological healthy response to hypothermia is high LF, HF as well as SDNN, RMSSD, and VLF. Studies performed in rodents and human adults without hypoxic-ischemic encephalopathy also found other HRV variables to increase with low temperatures^21,24,27^.

Previous studies used different frequency bands for analysis of frequency domain variables^15,17^. This reflects the limited consensus on measurement and interpretation of HRV in neonates. LF and HF has previously been interpreted as measures of sympathetic and parasympathetic activity^3^, but as no frequency bands has been agreed upon for neonates, we have been cautious not to make such interpretations.

Despite the influence of temperature, HRV recorded during therapeutic hypothermia may potentially be a measure to distinguish between favourable and unfavourable long-term outcomes after hypoxic-ischemic encephalopathy^11,17^. This could be by comparing HRV before and during hypothermia. Campbell et al. found the response in heart rate and blood pressure to routine-care event such as diaper change and physical examination to be reduced in newborns with moderate to severe encephalopathy^28^. In our neurophysiological healthy animals, the response to hypothermia was an increase in HRV. The response in HRV to hypothermia or other stimuli is of relevance to future research as the ability to regulate HRV during hypothermia could be of prognostic value.

## Limitations

Our study has limitations. The main limitation is the few animals involved in the study meaning that results should be interpreted cautiously. In accordance with the 3R’s principles (here; reduction and refinement) the animals were also used to study other outcomes^29^. We had no prior knowledge to inform a sample size calculation because previous studies were carried out in human newborns with a specific pathology, i.e., neonatal encephalopathy. Based on our previous experience (unpublished data) and the need for taking noise into account during ECG-recording, we estimated a priori that six animals would be sufficient for this particular study. The few animals included may increase the risk of both type 1 and 2 errors. However, we found an association between temperature and all, not just some, HRV variables. Thus, due this consistency within our findings, we consider the risk of accidental findings to be quite low. We calculated a post-hoc power of 82% based on 6 paired analyses of SDNN (corresponding to six piglets), with a difference in means of 27, a standard deviation of 16 and an alfa of 5%. The power is considered sufficient for this animal study.

We chose to focus on HRV only when the animals had stable temperatures during hypothermia and normothermia. In the clinical setting rewarming is slow and controlled, but the benefit of slow rewarming may be limited^30^. We cooled and rewarmed the animals as fast as possible. Future studies on the association between HRV and temperature at all temperatures from cooling to normothermia may be of clinical interest. We monitored temperature continuously but only registered every 15 min for analyses. While it is possible that temperature could change within these 15 min adjacent temperatures were similar, thus, it seems unlikely that unmeasured changes have affected our results.

Continuous infusion of anesthetics may result in accumulation and higher blood concentrations may influence HRV, in particular during hypothermia^31,32^. However, the infusion rate was reduced to a minimum in order to keep the animals sedated. The level of sedation was assessed regularly during the experiments. If HRV, however, was more suppressed by drugs during hypothermia, the influence of hypothermia on HRV measures would results in bias towards no difference between hypothermic animals and those with normothermia. To prevent biased results due to initial bolus of drugs during induction of anesthesia but also due to accumulation of drugs during continuous infusion, we designed the study as a cross-over study.

Animal 1 had a small decrease in HF and not as pronounced an increase in the other HRV variables during hypothermia as the other animals. ECG recording of Animal 1 was initiated shortly after intubation while the remaining animals started later. Thus, the bolus of anesthetics administered prior to intubation may have influenced HRV in Animal 1. Animal 1 had increasing HRV during the first hour of ECG recording when it was hypothermic while the other animals had more stable HRV values during the first hour of ECG recording when they were hypothermic. Thus, the bolus of anesthetics administered at intubation may not have been metabolized in Animal 1.

Potential interspecies differences in brain function complicate translation of our results to the clinical setting.

## Conclusion

Our results show that temperature affects HRV. Hypothermia was associated with an increase in all selected HRV variables. This could be mediated by bradycardia during hypothermia. Our study underlines the importance of considering temperature in future studies on HRV and hypoxic-ischemic encephalopathy; in order for HRV to be properly evaluated as a potential biomarker of brain injury.

## Methods

The experimental procedures were approved by and conducted in accordance with guidelines from the Danish Animal Experiments Inspectorate, which approved the study (2016-15-0201-01146). A protocol was prepared prior to the experimental procedures. The study is reported in accordance with the ARRIVE guidelines 2.0 (Checklist in Supplementary material)^33^.

The study was based on a neonatal piglet model previously used by our group^18,19,34^. Six healthy, term, Danish Landrace piglets, male and female, under 12 h of age, and weights between 1500 and 2000 g were studied. The animals came from a herd included in a health-monitoring program, which means the herd is screened for common pathogens that can affect pigs in a production setting. On each experimental day, two clinically healthy animals of the same sex, from the same litter, were received at the experimental facility directly from the farm.

### Anesthesia and monitoring

Anaesthesia was initiated with Sevoflurane inhalation (2–4%). Intravenous access was acquired by an ear vein and a bolus of Propofol (10 mg/kg), Fentanyl (30 µg/kg), and Rocuronium (1 mg/kg) was administered. The animals were then intubated and ventilated by pressure-control with FiO_2_ 21% at respiration rate 15–25 per minute to a target end-tidal CO_2_ of 4.5–5.5 kPa. After intubation animals were placed in the supine position. Anaesthesia was maintained throughout the experiment by continuous intravenous infusion of Propofol (4–10 mg/kg/h) and Fentanyl (5*–*12 g µg/kg/h). Level of consciousness were monitored continuously and anaesthesia was adjusted accordingly. Prophylactic Ampicillin (30 mg/kg) and Gentamycin (5 mg/kg) were administered intravenously once.

For additional intravascular access, arterial and venous umbilical catheters were inserted with aseptic techniques. The arterial catheter was used for arterial blood sampling and blood pressure monitoring. Continuous infusion of normal saline with Heparin (2 IU/ml) was used to ensure a patent arterial catheter. The venous catheter was used for continuous infusion (5–10 ml/kg/h) of NeoKNAG (Na^+^: 15 mmol/L, K^+^: 10 mmol/L, Cl^−^: 25 mmol/L, glucose: 505 mmol/L) to maintain blood glucose level and electrolyte- and fluid balance. Mean arterial blood pressure, O_2_ saturation, FiO_2_, and end tidal CO_2_ was monitored. Blood electrolytes, blood-glucose, pH, lactate, pO_2_ and pCO_2_ and standard base-excess was analysed from arterial blood samples taken every hour (ABL Radiometer Medical, Denmark). Fluid infusion and ventilator settings were adjusted accordingly.

### Temperature

After anesthesia and stabilization, the animals were subjected to hypothermia by active whole-body cooling with a cross-over design. Three animals were normothermic (38.5–39.5 °C) for the first 3 h and then subjected to hypothermia (33.5–34.0 °C) for 3 h. Three animals were hypothermic (33.5–34.0 °C) for the first 3 h and then rewarmed to normothermia (38.5–39.5 °C) for 3 h. A temperature of 38.5 to 39.5 °C is normal physiological core temperature for newborn piglets^18,20^. Hypothermia was initiated by active whole-body cooling using two bags of 5 °C saline water placed directly on the animal. During hypothermia the infusion rate of Propofol and Fentanyl was reduced to avoid accumulation due to reduced metabolism^35^. Rewarming was initiated using a heated inflatable air mattress. Temperature was monitored continuously with a thermometer placed approximately 5 cm into the rectum of the animal, i.e., animal core temperature. Temperature was measured continuously only during stable target temperatures while hypothermic and normothermic. Temperature was registered every 15 min.

After experimental procedures all animals were euthanized, while still anaesthetized, with Pentobarbital (400 mg/l, 0.2 ml/kg).

### ECG recording and HRV analysis

Electrodes for electrocardiogram recording (ECG) were placed on the right front limb and the two hind limbs. Continuous ECG was recorded throughout the experiment and computer logged at 300 Hz (Datex Ohmeda S/5 Collect, Finland) which corresponds to 300 datapoints on heart electrical activity per second^3,36^. HRV analysis was blinded to animal temperature. For each animal the raw ECG was uploaded to Kubios Premium (version 3.0.0)®^37^. Each R-wave was automatically identified by the software algorithm. Prior guess for RR-interval was fixed with average interval at 0.3–0.8 as suggested by Kubios®. This prior guess suggests a RR-interval length to the software prior to analysis^37^. Erroneous R-waves were corrected by manual identification. The ECG recording during 3 h of hypothermia and 3 h of normothermia was separated in successive, non-overlapping, 5 min epochs. This epoch duration is recommended by The Task Force of the European Society of Cardiology and the North American Society of Pacing and Electrophysiology, 1996^3^. Furthermore, the duration has been used in previous newborn animal and human studies of HRV^7,10,17^. A priori the following time domain and frequency domain variables were chosen as outcome. Time-domain variables were calculated automatically for each epoch and included: heart rate, SDNN, and RMSSD. Frequency-domain variables VLF, LF, and HF were automatically calculated using Fast Fourier Transformation and estimated as absolute power using Welch’s Periodogram. The following frequency bands previously used in newborns were applied for calculation: VLF: 0.02–0.04, LF: 0.04–0.2, and HF: 0.2–2.0^17,38^. We excluded epochs with: cardiac arrhythmia such as abnormal heart beats and artefacts due to noise or handling of the animal, e.g., ECG electrode replacement.

### Statistical analyses

Statistical analyses were performed using GraphPad Prism®V8 software and Stata/IC 16.1. Biochemical variables (blood-glucose, pH, lactate, haemoglobin, pO_2_, pCO_2_, standard base-excess) and MABP were averaged during hypothermia and normothermia. ∆T was defined as the positive change in temperature over 15 min. In two animals the temperature was only registered every 30 min. Thus, imputation with average of prior and subsequent observations was used for the missing values. Data are presented as mean with standard deviation (SD) for parametric data and median and interquartile range (IQR) for non-parametric data. The number of epochs, biochemical variables, MABP, and ∆T were compared between normothermia and hypothermia using paired t-test for normally distributed data or Wilcoxon test for non-normally distributed data. Normality was determined by visual inspection of the plotted data and by Shapiro Wilk test where normality was considered when the two-sided alfa-level was above 0.05.

HRV variables VLF, LF and HF were log-transformed. All HRV variables from all epochs were averaged to a mean covering 3 h of hypothermia and a mean covering 3 h normothermia. They were initially compared with paired t-test. A linear multiple regression model was used to investigate if there was effect of whether the animal was first exposed to hypothermia or normothermia. F-test was used to test the significance of temperature as categorical variable (normothermia or hypothermia) in this model where F < 0.05 was considered significant. To explore the association between HRV and heart rate and an association between fluctuations in temperature within the hypothermic interval and HRV we used linear regression analyses. As each animal contributed with multiple observations, we used clusters on each animal. A two tailed p-value of less than 0.05 was considered statistically significant.

## Supplementary Information

Supplementary Information.

## Data Availability

The datasets generated in the presented study are available from the corresponding author on request.
