# Supplementary material for: Hypothermia and heart rate variability in a healthy newborn piglet model
Source: Sci Rep. 2022 Oct 31;12:18282. doi: 10.1038/s41598-022-22426-3 (PMC9622714; doi:10.1038/s41598-022-22426-3)
Supplement: Supplementary file 1 — Supplementary Information. [file 41598_2022_22426_MOESM1_ESM.pdf]

The Recommended Set

These items complement the Essential 10 and add important context to the study. Reporting the items in both sets represents best practice.

Item		Recommendation	Section/line number, or reason for not reporting
**Abstract**	11	Provide an accurate summary of the research objectives, animal species, strain and sex, key methods, principal findings, and study conclusions.	2
**Background**	12	a. Include sufficient scientific background to understand the rationale and context for the study, and explain the experimental approach.	3
		b. Explain how the animal species and model used address the scientific objectives and, where appropriate, the relevance to human biology.	3
**Objectives**	13	Clearly describe the research question, research objectives and, where appropriate, specific hypotheses being tested.	3-4
**Ethical statement**	14	Provide the name of the ethical review committee or equivalent that has approved the use of animals in this study, and any relevant licence or protocol numbers (if applicable). If ethical approval was not sought or granted, provide a justification.	8
**Housing and husbandry**	15	Provide details of housing and husbandry conditions, including any environmental enrichment.	8
**Animal care and monitoring**	16	a. Describe any interventions or steps taken in the experimental protocols to reduce pain, suffering and distress.	8-10
		b. Report any expected or unexpected adverse events.	4
		c. Describe the humane endpoints established for the study, the signs that were monitored and the frequency of monitoring. If the study did not have humane endpoints, state this.	10
**Interpretation/ scientific implications**	17	a. Interpret the results, taking into account the study objectives and hypotheses, current theory and other relevant studies in the literature.	4-6
		b. Comment on the study limitations including potential sources of bias, limitations of the animal model, and imprecision associated with the results.	7
**Generalisability/ translation**	18	Comment on whether, and how, the findings of this study are likely to generalise to other species or experimental conditions, including any relevance to human biology (where appropriate).	5-6
**Protocol registration**	19	Provide a statement indicating whether a protocol (including the research question, key design features, and analysis plan) was prepared before the study, and if and where this protocol was registered.	8
**Data access**	20	Provide a statement describing if and where study data are available.	11
**Declaration of interests**	21	a. Declare any potential conflicts of interest, including financial and non-financial. If none exist, this should be stated.	12
		b. List all funding sources (including grant identifier) and the role of the funder(s) in the design, analysis and reporting of the study.	12
